# ﻿No end to endemism – contributions to the difficult *Nasa* Weigend Series *Alatae* (Loasaceae). A new species from Peru and the rehabilitation of “ *Loasa*” *calycina* Benth.

**DOI:** 10.3897/phytokeys.252.141635

**Published:** 2025-02-19

**Authors:** Tilo Henning, Joshua P. Allen, Daniel Montesinos-Tubeé, Eric F. Rodríguez-Rodríguez, José Luis Marcelo Peña, Rafael Acuña-Castillo

**Affiliations:** 1 Leibniz Centre for Agricultural Landscape Research (ZALF), Eberswalder Str. 84, 15374, Müncheberg, Germany; 2 Andes to Amazon Expeditions, Cuispes, Bongará, Amazonas, Peru; 3 Departamento de Ciencias Naturales, Universidad Católica San Pablo, Urb. Campiña Paisajista, s/n, Quinta Vivanco, Arequipa 04001, Arequipa, Peru; 4 Naturalis Biodiversity Centre, Darwinweg 2, 2333 CR Leiden, Netherlands; 5 Herbarium Truxillense (HUT), Universidad Nacional de Trujillo, Jr. San Martín 392, Trujillo, Peru; 6 Universidad Nacional de Jaén, Jaén - San Ignacio, 06800, Peru; 7 Escuela de Biología, Universidad de Costa Rica, Apdo. Postal 11501-2060, San Pedro de Montes de Oca, San José, Costa Rica; 8 Herbario Luis A. Fournier Origgi, Centro de Investigación en Biodiversidad y Ecología Tropical (CIBET), Universidad de Costa Rica, Apdo. Postal 11501–2060, San Pedro de Montes de Oca, San José, Costa Rica

**Keywords:** Amotape-Huancabamba Zone, Andes, Cajamarca, cloud forest, Cornales, iNaturalist, Loasoideae, narrow-endemic

## Abstract

A new species of Nasaser.Alatae (Urban & Gilg) Weigend from Northern Peru is described and illustrated. *Nasakatjae***sp. nov.** was at first encountered by an observation on iNaturalist and subsequently collected in the humid Andean forests near Colasay in the province of Jaén (Cajamarca, Peru). Whilst comparing the new species with closely related *Nasaloxensis* (Kunth) Weigend, a taxon widespread in Southern Ecuador (and tentatively adjacent Peru), a reevaluation of the status of earlier synonymized *Loasacalycina* Benth. became necessary. Consequently, *Nasacalycina***comb. nov.** is rehabilitated at species level and *Nasaloxensis* is redefined.

## Abstract (nl)

Describimos e ilustramos una nueva especie de Nasaser.Alatae (Urban & Gilg) Weigend del norte de Perú. *Nasakatjae***sp. nov.** fue inicialmente encontrada gracias a la plataforma de ciencia ciudadana iNaturalist y posteriormente recolectada en los bosques húmedos andinos cerca de Colasay en la provincia de Jaén (Cajamarca, Perú). Al comparar la nueva especie con *Nasaloxensis* (Kunth) Weigend, un taxón estrechamente relacionado y ampliamente distribuido en el sur de Ecuador (y posiblemente en Perú adyacente), fue evidente que una reevaluación del estado de *Loasacalycina* Benth., anteriormente sinonimizada, era necesaria. En consecuencia, proponemos la rehabilitación de *Nasacalycina***comb. nov.** a nivel de especie y redefinimos a *Nasaloxensis* basados en la evidencia acumulada.

## ﻿Introduction

Human expansion and technological advances have brought even some of the most remote natural habitats within immediate reach, while also exposing them to the imminent threat of destruction. As a result, new taxa are being discovered that often occur only in hard-to-reach, small-scale habitats, and most of them are threatened with extinction before they are even described (Brown et al. 2023). On a positive note, digitisation and the advent of online repositories have immensely facilitated and accelerated the initial report of taxa, increasing the chances that a taxon is discovered, scientifically described, and potentially protected before it disappears due to human caused habitat alteration and destruction (Henning et al. 2023). All of this came to fruition in the context of the discovery of a new species of *Nasa* Weigend (Weigend et al. 2006, Loasaceae subfam. Loasoideae), described here. It has a very narrow range and has very specific habitat requirements, making it vulnerable to human disturbance. The forest to which it is putatively endemic is unprotected and under pressure from agricultural expansion and climate change. A local Peruvian naturalist, Carlos Pérez Peña first reported the species photographically on the citizen science platform iNaturalist (http://www.inaturalist.org), it was then assessed by specialists, and subsequently collected and documented. The important efforts by interested citizens that make their discoveries available in public repositories, complement traditional herbarium, laboratory and field research carried out by specialists and have opened a new venue for scientists of all academic levels to document and improve the understanding of the enormous biodiversity in the tropics.

### ﻿Nasaser.Alatae (Urban & Gilg) Weigend

**Figure 1.**
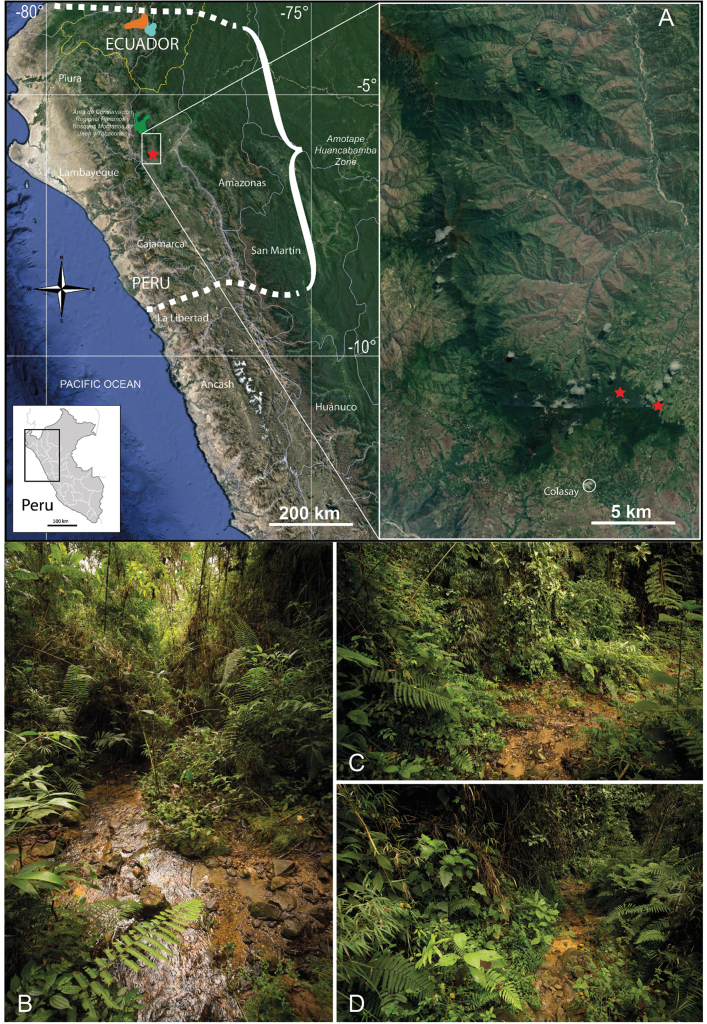
**A** Distribution of *Nasakatjae* and similar species. Left: Location of the forest system and the habitat of *N.katjae* (red star) in Peru and the Amotape-Huancabamba Zone, orange area: approximate distribution of *N.loxensis*, cyan area: *N.calycina*. Right: collections sites of *N.katjae* in the forest north of the town of Colasay **B–D** habitat of *N.katjae***B** small stream at “Agua fria” **C** path crossing a small stream at the type locality **D** flowering *N.katjae* among dense vegetation (left). Map data 2024 (C) Google. All photos by J. P. Allen.

Although revised only in 2000 (Weigend 2000a, 2000b, 2001), Nasaser.Alatae has repeatedly been expanded by new taxa since (Weigend 2004b; Rodríguez and Weigend 2004) due to continuous collection efforts, mostly in northern Peru. The series is restricted to four countries: Panama (1 sp.), Colombia (7 spp.), Ecuador (8 spp.) and Peru (19 spp.) and the species number has more than tripled from only 11 during the first half of the 20^th^ century (sensu Urban and Gilg 1900; Urban 1911; Macbride 1941, three additional names accepted at the time are currently synonymised under other names) to 35 within less than a decade (1996–2004). This sudden increase is due in part to Maximilian Weigend and his colleagues focusing on this group of plants and conducting targeted field campaigns, and in part to the fact that reachability and access to more remote forest areas is now much easier than in the past as mentioned before. Overall, Peru has 16 endemic species and, including the species also found in southern Ecuador, 17 species occur in the so-called Amotape-Huancabamba Zone (AHZ, Fig. 1A, Table 1), an area spanning from northern Peru into southern Ecuador (Fig. 1A), that is a known hotspot of biodiversity and is characterised by an extremely high rate of narrow endemism (Weigend 2002, 2004a) and is thus representing the most important area of distribution for this group.

**Table 1.** Currently accepted species considered part of Nasaser.Alatae. ** – the type species of the series; * – species of uncertain affiliation to the series. Species present in the Amotape-Huancabamba Zone (AHZ) are highlighted in bold.

Species	Distribution
***N.amaluzensis* (Weigend) Weigend**	Ecuador, Peru
***N.anderssonii* Weigend**	Ecuador, Peru
*N.auca* (Weigend) Weigend	Ecuador
*N.campaniflora* (Triana & Planchon ex. Urb. & Gilg) Weigend	Colombia
***N.carnea* (Urb. & Gilg) Weigend**	Peru
***N.dillonii* Weigend**	Peru
*N.dolichostemon* (Urb. & Gilg) Weigend	Colombia
***N.driessleae* Weigend**	Peru
***N.glabra* (Weigend) Weigend**	Ecuador
***N.lambayequensis* Weigend**	Peru
*N.lehmanniana* (Urb. & Gilg) Weigend	Colombia
*N.lenta* (J.F. Macbr.) Weigend	Peru
N.limata* (J.F. Macbr.) Weigend	Peru
***N.longivalvis* E. Rodr. & Weigend**	Peru
N.loxensis** (Kunth) Weigend (incl. Loasacalycina Benth.)	Ecuador
***N.nubicolorum* Weigend**	Peru
N.olmosiana* (Gilg ex J.F. Macbr.) Weigend	Ecuador, Peru
*N.panamensis* Weigend	Panama
N.pascoensis* Weigend	Peru
***N.pongalamesa* Weigend**	Peru
*N.profundiserrata* Weigend	Colombia
*N.puma-chini* (Weigend) Weigend	Colombia, Ecuador
*N.rubrastra* (Weigend) Weigend	Colombia, Ecuador
***N.sagasteguii* Weigend**	Peru
***N.solata* (J.F. Macbr.) Weigend**	Peru
***N.stolonifera* Weigend**	Peru
*N.tingomariensis* (J.F. Macbr.) Weigend	Peru
*N.trianae* (Urb. & Gilg) Weigend	Colombia
***N.urentivelutina* Weigend**	Peru
***N.victorii* Weigend**	Peru

**Figure 2.**
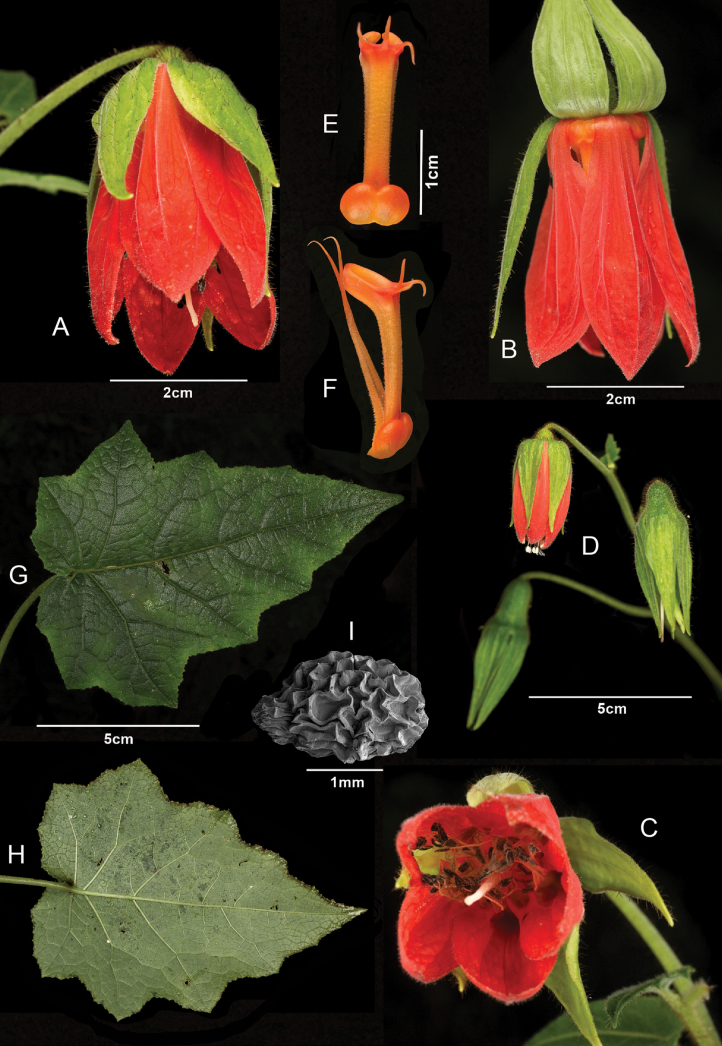
Lankester Composite Dissection Plate (LCDP) of *Nasakatjae***A** Flower, fronto-lateral view **B** flower, lateral view, sepals lifted **C** frontal view in late anthesis **D** inflorescence, not the elongated sepals on the young fruits **E** nectar scale, dorsal view **F** nectar scale lateral view with free inner staminodes **G** leaf adaxial surface **H** leaf abaxial surface **I** seed. Credit: **A–H** J. P. Allen **I** Y. Malkowsky.

Nasaser.Alatae, as originally defined, is morphologically comparably homogeneous. Species of this series were grouped in this taxon by Urban and Gilg (as LoasaAdans.sect.Loasa (≡ *Euloasa*) ser. Alatae) by virtue of their simple, pinnately veined leaves in combination with corollas with erect, often red or orange petals and very distinctive nectar scale wings bent 45°–90° relative to the nectar scale back (Gilg 1894; Urban and Gilg 1900; Fig. 2F). Due to these traits, recognizing a member of Nasaser.Alatae would be relatively straightforward, and some species could even be grouped in morphologically distinctive informal groups such as “carnea”, “lehmanniana”, “campaniflora” (Weigend 2000b; Weigend and Gottschling 2006). However, taxa within the same group are sometimes difficult to tell apart as their diagnostic characters are often relatively inconspicuous in herbaria, even if consistent. The comparatively few specimens of some taxa available in collections (something that is typical for *Nasa*: Henning et al. 2023) also make it difficult to assess the morphological variability of the species. Nasaser.Alatae includes many species adapted to wet premontane or montane forests (Weigend 2000a). An ecosystem rarely exploited by Loasaceae outside the tropical mountains, but also one that remains quite understudied due to difficulties in access and the complex topography of many mountain ranges, particularly the Andes. These are probably the reasons why assigning some specimens to accepted taxa often remains doubtful or impossible, despite recent and thorough revisionary works (Weigend 2000a, 2000b, 2001, 2004b).

The phylogenetic relationships of Nasaser.Alatae are not fully resolved. According to available molecular data, in particular plastid markers, most of the species currently considered as part of this section appear to belong in one of the four major clades of *Nasa* (Clade II in Acuña-Castillo et al. 2021). However, a few species traditionally included in this series (*Nasaolmosiana* (Gilg ex J.F.Macbr.) Weigend, *N.pascoensis* Weigend and, sometimes, *N.limata* (J.F. Macbr.) Weigend) seem to be more closely related to “non-*Alatae*” species, at least according to plastid data. To complicate matters, a group of morphologically very different taxa, traditionally placed in the large, plesiomorphic series *Saccatae* (Urban & Gilg) Weigend (e.g. *Nasasanchezii* T.Henning & Weigend) and the smaller series *Carunculatae* (Urb. & Gilg) Weigend (e.g. *Nasamacrothyrsa* (Urb. & Gilg) Weigend) have repeatedly been retrieved as nested within the same clade as the majority of Nasaser.Alatae spp. (Weigend and Gottschling 2006; Acuña-Castillo et al. 2021). Whether this is a result of past hybridisation events or convergent evolution is still unclear.

### ﻿The complex diversity of the group

*Nasa* is a relatively ancient genus (its crown age dating back to ca. 28 Ma), which probably originated in the proto–Central Andes, in mid-elevation, seasonally dry scrub habitats (Acuña-Castillo et al. 2019, 2021). Its history has been shaped by frequent dispersals and habitat shifts, especially in the AHZ which has acted mostly as a cradle for this genus (Acuña-Castillo et al. 2021). Nasaser.Alatae, which is primarily adapted to forest habitats, is often affected by habitat changes and (de)fragmentation in the dynamic landscape of the Andes, and this is especially noticeable in the very complex mosaic of habitats in the AHZ. As a result, the members of this group have a patchy and often very narrow distribution and tend to show morphological variability between different disjunct local populations. That is the case of our current concepts of *Nasaloxensis* and *N.olmosiana* and of *N.puma-chini* (Weigend) Weigend and *N.rubrastra* (Weigend) Weigend to a lesser extent. To add another layer of complexity, in the Central Cordillera of Colombia, the very different *N.trianae* (Urb. & Gilg) Weigend and *N.profundiserrata* Weigend could hybridize (Weigend 2001), so it is possible that other taxa may do the same where their ranges meet. The delimitation of species is therefore difficult, especially considering the incomplete sampling of suitable habitats in some of the areas with the highest diversity and the resulting limited herbarium evidence.

Besides a few clearly recognisable species such as e.g. *Nasadillonii* Weigend, *N.driessleae* Weigend and *N.urentivelutina* Weigend that are well characterised by single or a combination of stable striking morphological characters, many taxa seem to show a relatively broad intraspecific variability. Moreover, for many members of the group, the morphological spectrum is limited, and taxon delimitation often is based on evolutionary unstable character complexes that are prone to a certain degree of plasticity (e.g. petal shape and orientation, indumentum). Earlier taxonomic studies were based on the generally scarce herbarium material (cf. Urban and Gilg 1900), sometimes complemented by observations from a limited number of field trips (Macbride 1941). Thanks to the advent of freely accessible online repositories (e.g. iNaturalist), an ever-increasing number of photographic reports are now available. These low-threshold ways of making observations available across all levels of professionalism have become an invaluable and rapid source of information that complements the more sluggish traditional sources of evidence (Henning et al. 2023). We are conscious that repositories like iNaturalist also have their shortcomings (particularly in groups that are poorly curated, i.e. not critically assessed by specialists, or for observations where the diagnostic traits of an organism are not evident), and that they cannot substitute traditional herbarium collections and fieldwork. But, from our experience, observations that show diagnostic traits and that have been curated and evaluated by regional or international specialists of a certain group, have proven to be an extremely useful complement to more traditional, and more individualistic, field- and herbarium studies. To gather a similar volume of evidence as iNaturalist, would have taken weeks of fieldwork following months or years of planning. Consequently, to the already known diversity of Nasaser.Alatae, information is constantly added by new occurrence reports and geo-referenced photographic evidence. On the one hand, this leads to a consolidation of certain taxa whose morphological and geographical characterisation is confirmed by the growing evidence. On the other hand, for some taxa the morphological spectrum and, often in tandem, the known distribution is constantly expanding, often to a point where the original description becomes inadequate, and a new taxonomic assessment becomes unavoidable. It must be said that until this day, the overall picture is thus getting more and more complex and has not yet reached a point where species boundaries and distribution patterns become necessarily clearer for all taxa of the group. However, what is apparent is that the alpha-taxonomy of the group is not yet fully resolved and that the diversity and level of endemism as already indicated in earlier works (e.g. Weigend 2000a) appears endless. With every forest fragment that becomes accessible, new evidence is presented that constantly expands the morphological spectrum. To ultimately resolve the systematics of ser.Alatae, thorough molecular studies down to the population level will be needed. Unfortunately – and this is, in our opinion, the biggest shortcoming of online-only repositories – usually there is no physical sample created in the context of these photographic occurrence records, which would be an essential prerequisite for such an endeavor.

### ﻿The need for names

Peru has recently been determined as one of the global “darkspots” of plant diversity (Ondo et al. 2024) meaning that it is not only a hotspot of known phytodiversity, but also a region that harbors a large amount of expected unknown diversity that should be prioritised in order to determine the diversity and distribution of plant taxa. The assignment of this status is largely due to the large geographical share that Peru has in the Amotape-Huancabamba Zone with its exceptional rate of endemism and its diverse and small-scale habitats (see Henning et al. 2021 for references). The AHZ is one of the main regions that accounts for the Peruvian “areas housing most undescribed and poorly geolocated species” (definition “darkspots” of plant diversity – Ondo et al. 2024) that define the whole country as a darkspot of plant diversity.

Due to the urgent need for conservation in general and that of the remaining forest fragments of the Andes in the Amotape-Huancabamba Zone in particular, it is imperative to assess the respective local biodiversity. To put the logic of current protection mechanisms in simple terms: widespread taxa are less in need of protection than narrow endemics and, logically, we can only protect taxa that are validly described, preferably at the species level. And because time is running out, we cannot wait until the systematics of certain groups are solved by means of elaborate and lengthy molecular studies with their taxonomic consequences only subsequently resolved (if at all), which continues to take up valuable time. It would, however, also not make sense to hastily delimit and describe numerous aberrant local forms based on a single observation (given a type specimen is available) and thereby further complicate matters. But it is most desirable and urgent to describe taxa and thus make their names available for biodiversity assessments and potential conservation measures if their taxonomic status is apparent based on the (“good”) characteristics observed. In our opinion this is the case here. The new species *Nasakatjae* sp. nov. that we formalise below, shows a unique combination of characters that clearly and unambiguously separates it from the known taxa of the group. It is most likely a narrowly endemic taxon and thus makes it a valuable component of the flora of the unprotected forest patch it has been found in, which is increasingly threatened (see below). Consequently, *Nasakatjae* sp. nov. is described here as new to science and illustrated, its affinities with related taxa are discussed, all known aspects of its ecology are presented, and the known distribution and its potential threat status are detailed.

## ﻿Methods

### ﻿Plant collection

The type collection near Colasay in September 2023 was carried out based on the collection permit RD-000130-2023-DGGSPFFS-DGSPF.

### ﻿Revised material

We carried out extensive studies of herbarium specimens, or high-resolution images of herbarium specimens from the following herbaria (Thiers 2024): AAU, CHEP, E, F, GB, HA, HUT, K, LOJA, NY, P, QCA, US and USM. Additional studies were carried out on observations from iNaturalist.org.

### ﻿Conservation assessment

The tentative assessment of the conservation status of the *Nasa* species was made using the International Union for Conservation of Nature criteria (IUCN Standards and Petitions Committee 2024). For *N.loxensis* and *N.calycina* comb. nov., the area of occupancy (AOO) and the extent of occurrence (EOO) was calculated using GeoCat (Bachman et al. 2011). A cell width of 5 km was used to calculate the AOO. Since the suitable habitat of narrowly endemic *N.katjae* sp. nov. merely has the size of one grid, both values have been estimated according to the size of the forest fragment.

## ﻿Results and discussion

### ﻿Formal taxonomy

#### ﻿ Nasa katjae

Taxon classificationPlantaeCornalesLoasaceae

T.Henning, J.P.Allen & R.H.Acuña sp. nov.

E44E738B-F6F2-54E2-B7D0-D703436E03ED

urn:lsid:ipni.org:names:77356918-1

Figs 2 , 3C

##### Type.

Peru • Departamento Cajamarca, Provincia Jaén, Distrito Colasay, Above Colasay near Agua Fria. In dense forest, along the trail to Agua Fria in streams, climbing in the vegetation. Just below the peak at ca. 2500 m, -5.93990, -79.05400, 01. Sep. 2023, D. B. Montesinos 10003, T. Henning, J. P. Allen (Holotype: HUT No. HUT-64640!, Isotype USM).

##### Diagnosis.

The new species is morphologically most similar to *Nasacalycina* comb. nov. (see below) and differs from it in its very elongated stems, subscandent habit, proportionately broader leaf blades with a conspicuously deeply cordate base, sepals and petals almost twice as long (to 4 cm and 4.5 cm respectively), sepals and petals of equal length and nectar scales with 3 conspicuous apical dorsal threads up to 5 mm long.

##### Description.

Plants to 1.5–3 (–4) meters tall, covered with scabrid, and stinging trichomes, glochidiate trichomes restricted to the abaxial surface of the leaves along the veins. Stinging hairs (setae) scattered all over the plant but most densely on the stem, ovary, sepals (= fruit) and along the veins of the leaves. Apical parts of the petals set with few glandular hairs. Stems upright when young or growing in open areas, climbing through and leaning on adjacent vegetation when growing in dense undergrowth, base slightly woody. Leaves opposite, petiolate, petiole up to 13 cm long, leaf blades pinnately veined, 7–11 × 5–10 cm, widely ovoid to triangular, with 3–7 obtuse triangular lobes on each side, the lower ones up to 3 cm wide and 1.5 cm long, gradually decreasing in size towards the apex, the upper ones inconspicuous, margins serrate, each tooth with a hydatode, base conspicuously cordate (sinus to 1 cm deep). Inflorescence a monochasial or dichasial cyme, bracts alternate to 3 × 1.5 cm, one per flower, smaller than vegetative leaves, base shallowly cordate to truncate. Sepals 5, persistent, long acuminate, green, up to and 3.5–5 × 0.6–0.8 cm when fruiting, with 3 main veins, temporarily spreading in early anthesis, closely fitting on the petals later and further contracting in fruit. Petals 5, scarlet red, shallowly cymbiform, oblanceolate, base narrower than the limb but claw poorly differentiated, 3.5–4.5 × 1–1.5 cm, with 3 evident main veins, gaps between petals let the nectar sacs and scale bases visible when calyx lobes removed. Nectar scales 5, orange, 19–21 mm long and 5–6 mm wide at base, with 2 distinct, broadly ovoid, seemingly smooth (when fresh) nectar sacs at the base, each one as wide as nectar scale back (3 mm in diameter), nectar scale back rectangular, narrow 15 × 3 mm, straight papillose, margins with even longer papillae, ending in 3 conspicuous, distinct dorsal threads, inserted apically, up to 5 mm long and with two horizontal wings, seemingly smooth, 7 × 4 mm and diverging 90–120° from the back. Staminodes 2 per scale, c. 22 mm long, slightly sigmoid, base papillose, apex filiform. Stamens in 5 antepetalous fascicles with 10–20 each, anthers whitish when shedding pollen. Ovary broadly conical, with a rounded base, 5 × 5 mm, with 3 parietal placentae. Stigma lobes 3, decurrent on the style surface. Fruit a broadly clavate capsule with a globose base, 20–25 mm long (without sepals) and 8–10mm wide at sepal insertion, opening with three apical valves. Seeds numerous, ovoid, 2.5 mm long and 1.5 mm wide, testa black and reticulate.

##### Paratypes.

**Peru**, Cajamarca, Prov. Jaén, Dist. Colasay • Sector Aguas frias, bosque montano húmedo, 5°56'14.69"S, 79°03'19.33"W, 2542 m, 28 March 2024, *José Luis Marcelo-Peña, Marisela Rojas, Robert Zurita 11923* (ISV) • Ditto, 5°56'23.07"S, 79°03'14.28"W, 2550 m, 30 March 2024, *José Luis Marcelo-Peña, Marisela Rojas, Robert Zurita 12127* (ISV) • Ditto, 5°56'23"S, 79°03'14"W, 2550 m, 30 March 2024, *José Luis Marcelo-Peña, Marisela Rojas, Robert Zurita 12128* (ISV).

Photographic evidence (iNaturalist): **Peru**, Cajamarca, Prov. Jaén, Dist. Colasay • -5.92834, -79.05414, April 2022, *biomonstrando*, http://www.inaturalist.org/observations/143704890 (type locality) • -5.93739, -79.0351, December 2023, *biped_cub*, http://www.inaturalist.org/observations/194322389 • -5.93739, -79.04139, December 2023, *biped_cub*, http://www.inaturalist.org/observations/194324117.

##### Affinities.

The species appears to be morphologically closest to *Nasacalycina* comb. nov., a species endemic to a small area of southern Ecuador, in SE Loja and adjacent Zamora-Chinchipe. This taxon has been considered a synonym of the more northerly *Nasaloxensis* (Weigend 1996; Jørgensen and León-Yánez, 1999; Weigend 2000b), but detailed examination of their floral morphology showed that both taxa are best treated as closely related, but separate species (see below). The species can be found in forest edges and relatively open understories of wet montane “cloud” forest. Habit, leaf shape, perianth parts size and proportions, as well as size and insertion of the nectar scale dorsal threads separate the new species from *Nasacalycina* comb. nov. (Table 2), rehabilitated to species level in this article (see below).

**Table 2.** Comparison of the species most similar to *Nasakatjae* sp. nov.

	*Nasaloxensis*	*Nasacalycina*	*Nasakatjae*
max. plant height m	1.5	1	1.5–4
sepal : petal ratio	0.25–0.5 : 1	0.6–0.8 : 1	0.9–1.1 : 1
corolla shape	closed, tapered towards the apex	half open, cylindrical	half open to closed, campanulate to barrel-shaped
max. petal length × width mm	38 × 10	20 × 10	45 × 15
nectar scales length × width mm	11–16 × 3	10 × 3	19–21 × 5–6
dorsal threads on nectar scales	short (>1 mm) or absent	short (>1 mm) or absent	conspicuous to 5 mm long

Our first encounter with this species took place on iNaturalist (http://www.inaturalist.org/observations/143704890), where, at first, we suspected it was an unusual *Nasaloxensis* (a species whose concept has been quite broadly applied in the most recent revisions of Loasaceae of Ecuador: Weigend 1996; Jørgensen and León-Yánez 1999; Weigend 2000b). After a more thorough analysis, it is evident to us that both species could be quite closely related but have important and consistent differences that support the distinctiveness of this new taxon. However, the most striking difference that was impossible to assess in the iNaturalist observations, but obvious at the natural habitat, is the sheer size of the plants. Whilst a few plants along the path resemble the typical upright and medium-sized habit of most species of Ser. Alatae (e.g. *N.loxensis*), some specimens found off the path in dense vegetation, were leaning on and climbing through the dense undergrowth (e.g. bamboo). These plants can grow several meters long until they reach open areas to present their flowers freely, which are then presumably visited and pollinated by hummingbirds. Another striking character unique to the new species are the conspicuous elongated sepals. Although sepal length is subject to a large variation in many species of *Nasa* and usually of moderate systematic value, the characteristic sepals found in *Nasakatjae* are exceptional. They are as long as the petals and, in some cases, even longer and the persistent calyx closes after flowering and shows a further elongation (Fig. 2D). Whilst the function of the long calyx in fruiting remains unknown (possibly moisture regulation), the calyx lobes are visually striking during anthesis and greatly influence the floral display. They are initially nestled up to the petals and start spreading and bending out their apices with beginning anthesis, giving the flower an elegant, slightly bell-shaped appearance. The sepals (half-) spread in peak anthesis and bend back after the flower sheds and fruit development starts.

##### Etymology.

The new species is named after Katja Lohse, beloved partner of the first author, mother of their children and steady supporter of his scientific endeavors.

##### Distribution and ecology.

*Nasakatjae* has so far only been collected a few times. The type collection was made about 5 km north of Colasay (Cajamarca, Peru, Fig. 1A). It grows only in, and at the edges of small streams in the highest part of this isolated forest fragment (Fig. 1 B–D). It is associated with the typical floristic elements in these “bosques montanos húmedos” such as *Chusquea* sp., *Fuchsia* sp., *Vernonanthura* sp., *Saurauia* sp., *Miconia* spp. and *Viburnum* sp. The forest in the uppermost regions is largely intact and only disturbed by small paths and some recent, small-scale clearings. However, the increasing negative impact of livestock farming is evident from the cattle tracks visible everywhere along the paths and already within the forest fragments.

The plants are adapted to the very dense vegetation by a flexible growth habit. In the few more open areas where the path crosses the streams, the plants grow upright and begin to flower at a height of 1 to 1.50 m. In the wettest parts, where the dense primary vegetation consists mainly of impenetrable bamboo thickets (*Chusquea* sp.), the plants continue to grow in length until they reach an open area to display their flowers. Although difficult to measure, some individuals easily reach a size of more than 4 m. The flowers are typical “hummingbird-pollinated” flowers thus having open space around them is vital for successful pollination. All *Nasa* species are protandrous and exhibit a thigmonastic stamen movement and staggered stamen maturation (Weigend et al. 2010; Henning and Weigend 2012). In the taxa pollinated by hummingbirds, this movement is reduced to a minimum both geometrically (i.e. the angle the stamens bend) and temporally (the number of moved stamens = pollen packets in time). *N.katjae* sp. nov. has erect petals (in contrast to spreading petals in insect-pollinated taxa), therefore the stamen hardly move towards the center, and the pollen dispersal is limited to a successive maturation and dehiscence of the anthers. This is a secondary adaptation to the unreliable visitation probability and the random and long distances hummingbirds transport the pollen (Henning et al. 2018). However, we could not observe any visits to the plants. Ripe fruits with full seed set were found.

The new species must be considered narrowly endemic to the overall area. It has been found twice at a spring area called “Agua Fria” which is the type locality. Only very recently, two new observations have come to our knowledge that, according to the locality-data on iNaturalist, have taken place some approximately 2.5 km further east. However, it is very likely that the species follows the streams downhill through the forest and inhabits suitable areas within the primary forest in all directions.

##### Phenology.

The known populations have been visited four times now by different people between February 2023 and March 2024. All visitors report flowering and fruiting plants, and it can be assumed that the plants flower throughout the year without a substantial break reflecting the constant climate and minor seasonal variation in temperature and precipitation in this wet montane forest.

##### Preliminary conservation status.

The forest near Colasay represents the southeasternmost part of a larger forest system that is often referred to as the “Bosques Montanos de Jaén’’. A former protected area called “Área de Conservación Municipal de Bosque de Huamantanga’’ was recently expanded to become the “Área de Conservación Regional Páramos y Bosques Montanos de Jaén y Tabaconas” (D.S. N°005-2021-MINAM see https://www.gob.pe/institucion/sernanp/normas-legales/1896920-005-2021-minam, Fig. 1A) and covers almost 32000 hectares of the north-western parts of this forest system in the districts of Sallique, Chontalí and San José del Alto. Unfortunately, the southern district of Colasay and the surrounding forests are not yet part of this protected area. Although large areas are still intact, recent deforestation and the expansion of agricultural land are threatening the forests at higher altitudes, which not only harbor great biodiversity, but also represent an important watershed whose streams are the main tributaries for a number of important river systems. There is no evidence that *N.katjae* is present in other parts of the larger forest system. Given the peculiar habitat preference and the lack of other collections or reports of the species, even in a wider area, *N.katjae* appears to be very narrowly endemic. It presumably only occurs along a small number of streams in a few remote spots in the south-eastern part of the forest system and seems isolated from the other parts by the steep topography and its adaptation to flowing water. The forest patch north of Colasay has an extension of approximately 25–30 km^2^. It is only connected to the northwards running chain of forests in the west at lower altitudes that might represent a barrier for high-altitude taxa. The Extent of Occurrence (EOO) can thus be estimated as this single forest patch and the Area of Occupancy (AOO) is even smaller, and it appears possible that only a small number of populations exists at and near the type locality. Due to the visible expansion of agricultural activities from the lower towards the higher altitudes, the forest is being pushed back from all sides and is getting even more isolated from the other forests further north. Given these observations, the new species must be considered as Critically Endangered (CR) based on the criteria A3 (conditions *c* and *d*) B1 and B2 (conditions *a* and *b*) according to the IUCN guidelines (2024). Unlike in *N.loxensis* and *N.calycina* comb. nov., we did not use Geocat to calculate the EOO and AOO for *N.katjae*. Since the whole forest only has the size of one grid cell (25 km^2^) a comparison would be pointless and misleading. In fact, the de facto suitable habitat is much smaller, since only the uppermost parts of the forest appear moist enough.

### ﻿About the status and a new combination for *Loasacalycina* Benth.

#### ﻿ Nasa calycina

Taxon classificationPlantaeCornalesLoasaceae

(Benth.) R.H.Acuña & T.Henning comb. nov.

974C45A6-BC11-5E62-B946-7EE90AF4A345

urn:lsid:ipni.org:names:77356919-1

Fig. 3B

##### Basionym.

*Loasacalycina* Benth., Pl. Hartw. [Bentham]: 132. 1844.

##### Holotype.

Ecuador • [Prov. Loja] Mountains near Loxa, July [1841], C.T. Hartweg s.n. (Holotype: K barcode K000372874!)

##### Description.

Plants to ca. 1 meter tall, covered with scabrid, glochidiate and stinging trichomes, glandular trichomes inconspicuous or absent. Stems erect, cylindrical to ca. 1 cm diam. Leaves opposite, petiolate, petiole 2–4 cm, leaf blades pinnately veined, 6–15 × (2–)3–7 cm, narrowly ovoid to ovoid or triangular, with 4–8 lobes per side, in some leaves shallow and poorly defined, triangular, from wider than long to longer than wide, apices acute, the largest of a leaf 0.5–2.5 × 0.5–2 cm, the second or third usually the largest, becoming progressively smaller apically, margins serrate, each tooth with a hydatode, base cuneate, rounded or truncate, apex acuminate. Inflorescence a monochasial or dichasial cyme, 10–50 cm long, bracts alternate, one per flower, usually shorter and proportionally much narrower than the vegetative leaves, elliptical, 1.5–4 × 0.3–0.7 cm, diminishing in length, and particularly width, towards the inflorescence apex. with 4–7, broadly triangular, shallow, often indistinct lobes, base cuneate to rounded. Pedicels 1.5–2.5 cm long in anthesis, straight, often horizontal but ranging between 45° above or below the horizontal, the apex deflexed. Flowers deflexed, 3–6 per inflorescence branch. Sepals 5, narrowly triangular or ovate, apex acuminate, yellowish-green, 1.2–2 × 0.5–0.7 cm, with 3 main veins, as long or slightly shorter than the petals. Petals 5, orange red to carmine, shallowly cymbiform, oblanceolate, base narrower than the limb but claw short and poorly differentiated (to 4 mm wide), 1.5–2 × 0.6–1 cm, with 3 evident main veins, tip rounded or obtuse, basal gaps between petals leave the scale bases visible (gaps often not visible because they are either very small or hidden by the sepals), corolla more or less cylindrical, as wide basally as distally. Nectar scales 5, orange or red, 10 mm long and 3 mm wide at base, with 2 distinct, broadly ovoid, seemingly smooth (when fresh) nectar sacs at the base, each one as wide as the nectar scale back (ca. 2.5 mm in diameter), nectar scale back rectangular, narrow, 5.5 × 2 mm, straight, surface papillose, with 0, 2 or 3 short filiform threads, inserted subapically, < 1 mm long and with two horizontal wings, 3–4 × 1.5 mm. Staminodes 2 per scale, to 14 mm long, slightly sigmoid, narrowing apically, base papillose, apex filiform. Stamens in 5 antepetalous fascicles with 8–10 each, filaments to 12 mm long, anthers 1 × 0.5 mm, elliptical, cream before dehiscence, whitish when shedding pollen, black after pollen is shed. Ovary broadly conical to hemispherical, with a rounded base, ca. 5–6 × 5–6 mm, with 3 parietal placentae. Stigma lobes 3, shortly decurrent on the style surface, style to ca. 15 mm long. Fruit a broadly clavate to conical capsule on an erect pedicel, 3.5–5 cm long, with persistent sepals and a shortly tapering base, mature capsule 20–30 × 10–12 mm (width at sepal insertion), including an elongated conical apical projection, opening by the three apically dehiscing valves. Seeds not seen.

##### Notes.

**Figure 3.**
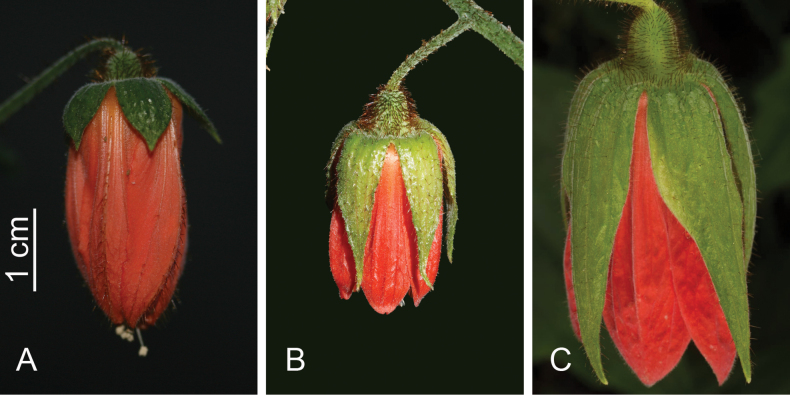
Comparison of flowers of *Nasakatjae* with its closest relatives in their actual size ratio **A***Nasaloxensis***B***Nasacalycina***C***Nasakatjae*. Photo credits: **A** M. Ackermann **B** R. Ripley **C** J. P. Allen.

*Loasacalycina* has been considered a synonym of *Nasaloxensis* since at least 1996 (Weigend 1996; Jørgensen and León-Yánez 1999). The most important differences between both species are evident in the proportions of the calyx and corolla elements with *N.calycina* having proportionally longer sepals, ca. ≥75% as long as the petals, while *N.loxensis* has sepals that are less than half the length of the petals (Table 2, Fig. 3). Also, the corolla in living anthetic flowers has a different shape in both species, as in *N.calycina* petals are arranged in a cylindrical shape with a proportionally wide corolla opening, while in *N.loxensis* the corolla tapers gradually, so distally the corolla is narrower than basally, with the petals leaving a very narrow opening (Fig. 3A, B).

In the latest and most authoritative treatment of Loasaceae of Ecuador, Weigend (2000b) discussed briefly the significant morphological variability of his broad concept of *Nasaloxensis*. But he recommended maintaining it until either more specimens from areas became available or it became clear that few or no additional specimens were known.

Weigend (2000b) acknowledged the differences in floral proportions of some specimens from Loja (that we consider fit well under the concept of *Loasacalycina* of Bentham) from those of Azuay (e.g. near Cuenca and Cajas National Park) that conform better to Kunth’s concept of *Loasaloxensis*. However, it is important to note that the type material of both *Nasaloxensis* and *Nasacalycina* come from near the city of Loja, where still today plants that adjust very closely to these specimens grow. The wider availability of more specimens in the mosaic of habitats (from pretty much pristine to significantly anthropogenically modified) around the city of Loja, and further north, along with photographs of living plants in platforms like iNaturalist have allowed us to confirm the relatively modest but seemingly consistent morphological differences between *Nasaloxensis* and *N.calycina*. The lack of florally intermediate specimens in possible areas of contact near Loja suggests that hybridisation is so infrequent, that truly intermediate specimens have not been recorded so far. From the specimens and photographs available we know that both species approach each other but appear to be sympatric in only one locality, between La Argelia and La Palma, SW of the city of Loja, and no evident intermediates in floral characters have been observed. Living plants that fit closely Hartweg’s type material of *Loasacalycina* have been collected most frequently from what is now Podocarpus National Park

GenBank accessions AY285722.1, MK333044.1, MK333010.1, MK332976.1, MK332944.1 and AY769214.1 identified as belonging to *Nasaloxensis* and used in the studies of Weigend et al. (2004), Weigend and Gottschling (2006) and Acuña-Castillo et al. (2019, 2021) belong to *Nasacalycina* (voucher: *J.R. Grant & L. Struwe 01-4063*).

##### Specimens examined.

**Ecuador**. Loja • Carretera Loja-Yangana, desvío al Parque Nacional Podocarpus, E del Nudo de Cajanuma, 2880–3000 m, 14 March 1989, *A. Freire-Fierro 1323* (GB, QCA) • Parque Nacional Podocarpus, E of Nudo de Cajanuma, hectare plot near “Centro de información”, c. 2900 m, 15 April 1989, *B. Eriksen 91176* (QCA) • Parque Nacional Podocarpus sector Cajanuma, 2900 m, 30 June 1996, *P. Lozano 449* (Loja) • Cajanuma, 21 February 1985, *F. Vivar 2310* (Loja) • Parque Nacional Podocarpus, above Nudo de Cajanuma, 2800–3000 m, 14–15 May 1988, *B. Øllgaard et al. 74090* (AAU, Loja) • Parque Nacional Podocarpus, S of Loja, wet montane forest at the “Centro de Información”, E of Nudo de Cajanuma, 2850–1950 m, 21–22 February 1985, *B. Øllgaard et al. 57847* (AAU, Loja) • Cajanuma, 04°06'24"S, 79°11'22"W, 2600 m, 31 January 2002, *P. Lozano et al. E-600* (Loja) • Entrada a Cajanuma, sendero a las Lagunas, 3100 m, 11 June 2002, *P. Lozano et al. E-1650* (Loja) • Uritushinga, 2800 m, 14 February 1978, *F. Vivar 898* (Loja) • Sitio Ventanas, Cordillera Oriental, Or. Loja, ca. 2400 m, 27 December 1947, *R. Espinosa & A. Espinosa 2279* (Loja) • Road La Argelia (southern Loja)-La Palma, along crest of mountain range just SW of Loja, 2700–2950 m, 4 March 1989, *B. Øllgaard et al. 90792* (AAU, Loja) • Zamora-Chinchipe: Parque Nacional Podocarpus (San Francisco entrance); trail leading west from San Francisco, 03°59'24"S, 079°05'48"W, 2100 m, 16 February 2001; *J.R. Grant & L. Struwe 01-4063* with *C. Rosales* (Loja, QCA) • Área del Parque Nacional Podocarpus, Cajanuma, rotundamente “ El Mirador”, 3000 m, s.d., *Rbu & SL 107* (QCA).

Photographic evidence (iNaturalist): (Note: due to *Nasaloxensis* s.l. being considered threatened by the IUCN, it was not possible to obtain the precise locality of some observations): **Ecuador**. Loja • -4.113259,-79.174943, April 2024, *Daniel Arias-Cruzatty*, http://www.inaturalist.org/observations/2057414 • April 2022, *Lilia Cueva Cueva*, http://www.inaturalist.org/observations/114005635 • December 2020, *prengelv*, http://www.inaturalist.org/observations/67754076 • Parque Nacional Podocarpus, Cajanuma, sendero a las Lagunas del Compadre, cerca del páramo, December 2020, *Angel Hualpa Erazo*http://www.inaturalist.org/observations/67015645 • Parque Nacional Podocarpus, Cajanuma, December 2020, *Amarú Ramóm Salcedo*, http://www.inaturalist.org/observations/66984658 • 04°07'3.34"S, 079°09'53.21"W, 3088 m, February 2017, *dennisronsse*, http://www.inaturalist.org/observations/37683383 • Near Cajanuma Refuge, September 2007, *Ruth Ripley*, http://www.inaturalist.org/observations/35868409 • ditto, http://www.inaturalist.org/observations/35868095 • ditto, http://www.inaturalist.org/observations/34983697 • ditto, http://www.inaturalist.org/observations/34981561 • Cerca del Refugio Cajanuma, -4.115681 -79.171616, February 2019, *Bodo Nuñez Oberg*, http://www.inaturalist.org/observations/20486169 • Cajanuma Field Station, and trail to Mirador, 04°06'45"S, 079°10'37"W, December 2007, *Jason Grant*, http://www.inaturalist.org/observations/20486169.

##### Distribution.

*Nasacalycina* is endemic to Loja and Zamora-Chinchipe (Fig. 1), mostly known from areas near or within Podocarpus National Park (mostly around the Cajanuma sector), where *Nasaloxensis* is missing. There is a single record between La Argelia and La Palma in an unprotected area where it seems to meet the southernmost range of *Nasaloxensis*. Further south in Loja (and reaching Piura, Peru), both taxa seem to be replaced by *Nasaamaluzensis*, a species that is florally quite close to *Nasacalycina* but differs significantly in leaf morphology.

##### Phenology.

Flowering has been recorded during September and mostly from December to July.

##### Tentative conservation assessment.

The known range of *Nasacalycina* is diminutive, but most of the populations of the species appear to be protected within the Podocarpus National Park, where it is frequently encountered in the Cajanuma sector. We still recommend considering this species as EN B2abiii, due to a reduced AOO (125 km^2^), the reduced number of known locations where the species can be found (only four) and the inferred decline of habitat quality where the only known population not found within or in the immediate vicinity of Podocarpus National Park grows.

#### ﻿ Nasa loxensis

Taxon classificationPlantaeCornalesLoasaceae

(Kunth) Weigend in Weigend et al. Revista Peru. Biol. 13(1): 77. 2006.

E4AA965F-33C7-5024-B281-AD970B4951B0

Fig. 3A

##### Basionym.

*Loasaloxensis* Kunth, Nov. Gen. Sp. 6: 116.1823.

##### Holotype.

Ecuador • [Prov. Loja] Loxa, [Aug?. 1802], A.v. Humboldt & A. Bonpland 3349 (Holotype: P barcode P00679495!)

##### Description.

Plants to ca. 1.5 m tall, covered with scabrid, glochidiate and stinging trichomes, glandular trichomes inconspicuous or absent. Stems erect, cylindrical, to 1 cm diam., base woody. Leaves opposite, petiolate, petioles 1–6 cm long, leaf blades pinnately veined, 3–14 × 2–8.5 (–11.5) cm, triangular to ovate, with 1–6 lobes per side, widely triangular to ovate, apices acute or rounded, the largest of a leaf, 0.5–2(–4.5) × 0.4–2(–3) cm, the first or second often the largest (the third less commonly so), progressively smaller apically, margins serrate to denticulate, each tooth with a hydatode, base rounded, truncate or shallowly cordate (sinus to 5 mm deep), apex acuminate. Inflorescence a monochasial or dichasial cyme, 7–30 cm long, bracts alternate, one per flower, usually shorter and proportionally much narrower than the vegetative leaves, elliptical, 1–8 × 0.2–3.2 cm, diminishing in length, and particularly width, towards the inflorescence apex with 4–7, broadly triangular, shallow to very shallow lobes, base rounded. Pedicels 2.5–3.6 cm long in anthesis, often horizontal but ranging to 45° above or below the horizontal, the apex deflexed. Flowers deflexed, up to 7 per inflorescence branch. Sepals 5, ovate triangular, apex acute to acuminate, green, 0.7–1.3 × 0.4–0.7 cm, with three main veins, evidently much shorter than the petals. Petals 5, orange to red, shallowly cymbiform, base narrower than the limb but claw short and poorly differentiated (to 4 mm wide), 2.5–3.8 × 0.8–1 cm, with 3 evident main veins, tip acuminate, basal gaps between petals leave the scale bases visible (gaps often visible, sepals too short to hide them), corolla wider at base, tapering distally. Nectar scales 5, yellow to orange red, going from lighter yellow on the back to darker orange on sacs and red orange on neck, 11–16 mm long and ca. 3 mm wide at the base, basally on back with two depressed globose sacs 2 mm in diam., nectar scale back rectangular narrow, papillose, dorsal threads 0, 2 or 3, short, < 1 mm long (very rarely longer), inserted subapically, scale neck thickened, slightly recurved, laterally protracted into two horizontal wings 5 mm long and 1 mm wide. Staminodia 2 per scale, 17 mm, narrowing apically, apex filiform, more or less sigmoid, slightly papillose, white. Stamens, usually extending beyond the petals during the male phase, in 5 antepetalous fascicles of 13–16 each, filaments to 30+ mm long, anthers 1.5 mm long and 1 mm wide, elliptical, cream before dehiscence, whitish when shedding pollen, black after pollen is shed. Ovary broadly conical or hemispherical, with a rounded base, to 7 × 5–6 mm, with 3 parietal placentae. Stigma lobes 3, shortly decurrent on the style surface, style to ca. 30 mm long. Fruit a clavate or cylindrical capsule with persistent sepals, pedicel erect, 25–55 mm long, mature capsule 21–27(–30) x 11–13(–14) mm (width at sepal insertion) including a short or elongated conical apical projection, opening by the three apically dehiscing valves, base rounded, less commonly tapering. Seeds numerous, dark brown, ovoidal, testa reticulate.

##### Notes.

For detailed information on identification, ecology etc. see notes under *Nasacalycina*. Across its distributional range, our redefined *Nasaloxensis* seems to show high uniformity in floral characters, although the observed leaf morphology can be significantly more diverse. The most unusual (and variable) leaf morphologies have been reported from the southernmost populations directly to the west and south of the city of Loja (Weigend 2000b). Some plants in this area have leaf blades with very prominent, long lobes and deep sinuses, and are occasionally as wide as long. The reasons for the high foliar variability in this part of the range are unknown. Living plants that closely resemble the type material of *Nasaloxensis* (*Humboldt & Bonpland 3349*) have been collected from localities to the west and north of the city of Loja.

##### Specimens examined.

**Ecuador**. Cañar • Parroquia Rivera, sector Santo Tomás, vía a Monay, propiedad de Luis Méndez, 2754 m, 12 October 2000, *A. Verdugo et al. 230* (HA) • Azuay Sevilla de Oro, 10–12 km N of village, 2750–2850 m, 11 September 1976, *B. Øllgaard & H. Balslev 9331* (AAU) • The Eastern Cordillera, 1–8 km north of the village of Sevilla de Oro, 2400–2700 m, 27 July – 12 August 1956, *W. Camp E-4339* (P) • Gualaceo a Macas, a 12 km de Gualaceo, 6 August 1986, *A. Freire-Fierro 251* (GB) • West of Patul 3 km between Huahualcay and Río Patul below Pasas de Pinglión, 2670–3275 m, 19 May 1943, *J. Steyermark 52611* (F) • Mountains near Cuenca, August 1864, *W. Jameson s.n.* (E, US) • Rio Machangara, NW Cuenca, quebrada vegetation, 3000–3100 m, 18 September 1967, *B. Sparre 18609* (US) • Sayausid, ca. 3000 m, 1 April 1968, *G. Harling et al. 7942* (GB) • Mountains above Sayausid, 3000–3200 m, 18 March1974, *G. Harling & L. Andersson 12604* (GB) • Above Sayausí, at first bridge over Río Tomebamba. Secondary scrub, 3200 m, 3 March 1985, *G. Harling & L. Andersson 22702* (GB, QCA) • Above Sayausí, trail to Cajas, 3300 m, 20 July 1939, *C. Penland & R. Summers 1074* (F, US) • Río Mihuir (Miguir?) 1 km below Miguir on road Cuenca – Molleturo. Secondary scrub, 3400 m, 8 March 1985, *G. Harling & L. Andersson* 22915 (GB, QCA) • Cuenca, Cajas, Laguna Llaviuco (=Laguna Surocucho). Directly on the roadside, 3000–3150 m, September – October 1995, *M. Weigend & S. Horn 3830* (F, QCA) • Area Nacional Recreacional Cajas, Sect. Llaviuco, 3300 m, 8 January 1991, *S. León et al. 2509* (QCA) • Surucucho, 2800 m, March 1967, *F. Vivar et al. 514* (Loja) • Cuenca, Sayausí, sector Dudahuayco, captación de agua. En sitios abiertos y pastizales, 3060 m, 20 July 2006, *A. Verdugo & D. Minga 1678* (HA) • Cuenca, Sayausi. Road to Llaviucu lagoon, close to the intersection with the Cuenca-Molleturo road. Common in degraded vegetation on the roadside, 3024 m, 18 February 2017, *R. Acuña & H. Garzón 1732* (QCA) • Las Cajas: near Laguna Llaviuco Montane forest and disturbed areas along the road, 3100–3200 m, 12 September 1983, *B. Boysen Larsen & B. Eriksen 45098* (AAU) • Parque Nacional Cajas, Laguna de Llaviuco, 3170 m, 9 June 2011, *C. Ulloa et al. 2137* (HA, QCA) • Fierroloma, Zorrocucho, en bosque primario, 3200 m, 15 January 1997, *D. Minga 86* (HA) • Dudahuaycu, Mazán, 3500 m, 6 March 1991, *G. Chacón 93* (HA) • 14 July 1994, *G. Chacón 94* (HA) • A 1 km del control de la vía a Loja, partidero del lado derecho hacia Yanasacha, bosque secundario y pajonal, zona lluviosa, 3000–3200m, 26 June 1978, *J. Jaramillo & J. Boeke 412* (QCA) • Río Matadero valley near entrance to Parque Nacional de Cajas, 2900 m, 28 December 1979, *L. Holm-Nielsen 20928* (AAU) • Sunsun-Yanasacha. Vía a mina de Caolín, cerca el río. En el sotobosque, 3100 m, 16 June 1999, *F. Serrano et al. 718* (HA) • Vía partidero a Quinoas-Surocucho, 3000 m, 5 February 1978, *F. Ortiz & J. Jaramillo 76* (QCA) • Portete del Tarqui and environs, remnants of montane forest, 2600–2700 m, 24 February 1993, *G. Harling & B. Ståhl 26666* (QCA) • Tarqui, near the monument, 2600 m, 5 February 1982, *G. Harling et al. 20247* (GB) • Victoria del Portete, sector Aguarongo y/o Caspishitana. En el sotobosque, 3584 m, 26 April 2006, *A. Verdugo & D. Minga 904* (HA) • 15 km SW of Cuenca on road to Giron, 8 km SE on dirt road from Hacienda Tarquí turnoff, km 22, to Patococha, 2950 m, 30 May 1990, *P. Peterson & E. Judziewicz 9359* (QCA) • Patacocha, 7–8 km by trail S of Hacienda Tarqui at Inquis, 3050–3100 m, 29 January 1988, *U. Molau et al. 2750* (GB) • Victoria del Portete, Río Portete, sector captación de agua, 2778 m, 25 April 2006, *A. Verdugo & D. Minga 887* (HA) • Sigsig to Gualaquiza, Rio Altarurcu (20 km E of Sigsig), 2800 m, 13 April 1968, *G. Harling et al. 8309* (GB) • Cumbe, 2900 m, 22–24 April 1968, *G. Harling et al. 8692* (GB) • 13 km S of Cumbe, 3300 m, 9 Jun 1979, *B. Løjtnant et al.14417* (AAU, GB) • Along Pan-American Highway, 40 km south of Cuenca, 3300 m, 20 September 1944, *I. Wiggins 10761* (US) • Loja: Saraguro, camino Panamericana-Huashapamba, montaña húmeda, 2800–3000 m, 31 September 2004, *A. Macas s.n.* (CHEP) • Vicinity of Las Juntas, 28 September 1918, *J. Rose et al. 23189* (US) • Between La Toma and Loja, 1800–2600m, 4 September 1923, *A. Hitchcock 21370* (US) • Road Catamayo (La Toma)-Loja, km 9 past junction with old road. Bosque húmedo remnants and secondary scrub, 2550 m, 28 April 1997, *G. Lewis 3216* (E, Loja) • West of Loja, just over pass to Catamayo (Toma); 1 km south of Loma de Trigal, scrub forest, 5 May 1986, *M. Baker 6959* (NY, US) • Cerro Villonaco, 2600–2750 m, 12 April 1974, *G. Harling & L. Andersson 13461* (GB) • W Slope, 10 February 1985, *G. Harling & L. Andersson 21862* (GB, QCA) • 1 March 1947, *R. & A. Espinosa 1316* (Loja) • Cerro Uritusinga. Loja-La Palma, km 18–20. Montane forest, primary and secondary forest under heavy pressure, exploited to produce charcoal, 2910–3000 m, 30 November 1994, *P. Jørgensen et al. 1040* (Loja) • Loja, 8 November 1876, *E. André K-546* (F) • Parte alta de Hacienda Montecristi, unos 40 km NE de Loja, curso del Río Zamora hacia el oriente, 8 June 1947, *R. Espinosa 1471* (Loja) • Cantón Catacocha, El Almendral, Hacienda La Hamaca, 1800–2200 m, 16 April 1944, *M. Acosta Solís 7902* (F) • Chepel, 2200 m, s.d., *R. Espinosa 1978* (Loja) • Prov. Unknown: Herb. de Pérou, s.d. *J. de Jussieu s.n.* (P) • Équateur et Pérou, s.d., *M. Grisar s.n.* (P) • *Warszewicz s.n.* (F neg, No. 10202).

Photographic evidence (iNaturalist): (Note: due to *Nasaloxensis* s.l. being considered threatened by the IUCN, it was not possible to obtain the precise locality of some observations): Ecuador • Azuay: -2.98455, -79.07776, April 2023, *Kabir Montesinos*, http://www.inaturalist.org/observations/154122854 • Laguna Sorocuchu, -2.84466, -79.14866, April 2023, *Kabir Montesinos*, http://www.inaturalist.org/observations/153135367 • W del P.N. Cajas, -2.89167, -79.36357, May 2021, *Kabir Montesinos*, http://www.inaturalist.org/observations/80194774 • Cerca del Portete de Tarqui, -3.08651, -79.13816, March 2024, *Kabir Montesinos*, http://www.inaturalist.org/observations/204670994 • Cerca del Portete de Tarqui, -3.08931, -79.13622, November 2022, *Kabir Montesinos*, http://www.inaturalist.org/observations/141022114 • Carachula, -3.14969, -79.35685, June 2023, *Kabir Montesinos*, http://www.inaturalist.org/observations/167995363 • Loja: Washapamba, -3.671097, -79.252418, May 2023, *LostInCR*, http://www.inaturalist.org/observations/178891174 • camino de Washapamba a Cerro de Torre-3.682952, -79.244846, April 2022, *Rudy Gelis*, http://www.inaturalist.org/observations/118712124 • Province Undetermined: Aug.2019, *manuelganzhi*, http://www.inaturalist.org/observations/31622038 • July 2020, *Jonathan Aguirre Pesantez*, http://www.inaturalist.org/observations/67707816 • June 2022, *Jonathan Aguirre Pesantez*, http://www.inaturalist.org/observations/121346890 • September 2022, *bb_593*, http://www.inaturalist.org/observations/134805560 • October 2022, *bb_593*, http://www.inaturalist.org/observations/137584820 • February 2022, *Edgar Segovia*, http://www.inaturalist.org/observations/106356391.

There are two observations from southern Cajamarca in Peru that are tentatively placed here. Since no herbarium material was available so far, we refrain from reporting a Peruvian distribution of this species for the time being. **Peru** • Cajamarca, -6.47573, -79.01225, August 2022, *manuelroncal*, http://www.inaturalist.org/observations/131296479 • -6.23583, -79.07411, August 2007, *barbetboy*, http://www.inaturalist.org/observations/15334620.

##### Distribution.

*Nasaloxensis* has a wide distribution in southern Ecuador, found in the provinces of Cañar, Azuay, Loja and apparently Morona-Santiago (Fig. 1). It is one of the most frequently collected species of *Nasa* in Ecuador as it can be found growing near human settlements, frequently visited national parks, and in both pristine and degraded habitats. At its southernmost distribution limits, it is suddenly replaced by *Nasacalycina*, farther to the SW, *Nasaamaluzensis* replaces both taxa. Further north in Tungurahua and Cotopaxi, similar *Nasaauca* can be found. As indicated above, a disjunct distribution at the southern end of the Amotape-Huancabamba Zone in Peru (Cajamarca, Prov. Chota) appears possible but needs further examination and material.

##### Phenology.

Flowering has been recorded every month of the year.

##### Tentative conservation assessment.

We recommend considering this species as NT. Although both its AOO (900 km^2^) and EOO (12.193 km^2^) could suggest a Vulnerable (VU) status like Cornejo and Suin (2011) recommended previously, these seem to be the only criteria that could justify such assessment as no other criterion appears to support a high-risk category. *Nasaloxensis* can be seen frequently in the W and S of the city of Cuenca and seems to be able to withstand some habitat degradation. Seemingly healthy populations are protected within the current limits of Cajas National Park.

### ﻿Key to the species of Nasaser.Alatae present in the Amotape-Huancabamba Zone (modified and extended from Weigend 2004b)

**Table:** 

1	Calyx tube and fruit without stinging hairs or, if with them, few, unevenly distributed and mostly restricted to base of the ovary/fruit	**2**
–	Calyx tube and fruit usually densely covered with stinging hairs, these evenly distributed across the outer surface of the ovary/fruit	**4**
2	Plants with evident stinging hairs; stems always lacking prominent ridges; mature capsules clavate, base acute	***N.anderssonii***
–	Plants mostly without evident stinging hairs; stems, at least when young, with prominent ridges; mature capsules subglobose to broadly cylindrical, base rounded	**3**
3	Petals ovate, narrowing gradually distally; leaf lobes usually obvious	***N.glabra***
–	Petals obovate, broadly rounded distally; leaf lobes often not obvious	***N.victorii***
4	Leaves pentagonous with acute leaf lobes; stem densely covered with uniseriate glandular hairs, especially in distal portion; stinging hairs to 5 mm long	***N.amaluzensis***
–	Leaves ovate to widely ovate, if widely ovate then leaf lobes always rounded to acuminate; stem without or with relatively few uniseriate glandular hairs; stinging hairs typically under 4 mm long	**5**
5	Corolla yellow; leaves with white lines along primary and secondary veins adaxially, lamina dark green	***N.driessleae***
–	Corolla pink, orange, or red, never yellow; leaves without white lines along veins or, if with white veins, then lamina bright green	**6**
6	Inflorescence with large, sessile, semi-amplexicaul bracts	***N.olmosiana***
–	Inflorescence with petiolate or sessile bracts, never semi-amplexicaul	**7**
7	Lamina very densely pubescent, velvety to the touch with numerous stinging hairs between the trichomes; vegetative shoots with numerous, mostly alternate, spirally inserted leaves (rarely opposite); stiffly erect, sparsely branched shrub	***N.urentivelutina***
–	Lamina hairy and sometimes densely so, but never velvety to the touch; leaves never alternate, always opposite; plant often branched from base	**8**
8	Dark green calli below insertion of petiole present; petals either very narrow (5–6 × as long as wide) or widely ovate, carnose and completely lacking stinging hairs on back; capsule clavate; pedicel elongating post-anthetically	**9**
–	Dark green calli below insertion of petiole absent, petals not narrow (< 4 × as long as wide) nor carnose; capsule variable in shape; pedicel not elongating post-anthetically	**11**
9	Petals ovate (< 4 × as long as wide), carnose	***N.sagasteguii***
–	Petals linear (5 > 6 × as long as wide), membranaceous	**10**
10	Leaves ovate, up to 90 × 50 mm; Peru: Piura	***N.solata***
–	Leaves widely ovate to subcircular, 90–150 mm long and wide; Peru: Cajamarca	***N.dillonii***
11	Petals half-spreading and corolla star-shaped; floral scales white or pale yellow	**12**
–	Petals erect and corolla tubular to campanulate; floral scales white, pale yellow, orange, red, or deep pink	**14**
12	Sparsely branched, stiffly erect herb; petals pink; Peru: Cajamarca	***N.carnea***
–	Much-branched stoloniferous herb or sub-shrub; petals orange or reddish-pink; Peru: La Libertad (Bolívar) south to Áncash	**13**
13	Stem and leaf veins with numerous yellow to red stinging hairs and scabrid hairs; petals orange, narrowly oblong, slightly cymbiform; capsule apical valves much shorter than the capsule below sepal insertion	***N.stolonifera***
–	Stem and leaf veins mostly without stinging hairs but with scabrid and glochidiate hairs; petals reddish-pink, narrowly ovate, deeply cymbiform; capsule apical valves about as long as the capsule below sepal insertion	***N.longivalvis***
14	Petals pale pink	***N.pongalamesa***
–	Petals orange or red, never pale pink	**15**
15	Petals narrowing strongly basally (claw well differentiated), leaving most of the nectar scales (both base and back) exposed; growing in Jalca	***N.lambayequensis***
–	Petals narrowing weakly basally (claw poorly differentiated), usually hiding most of the nectar scale (only the bases exposed), growing in montane forest and subpáramo	**16**
16	Petals widely ovate, half-spreading; corolla campanulate; Peru: Amazonas	***N.nubicolorum***
–	Petals oblong, oblanceolate to narrowly ovate, erect; corolla conical, tubular to narrowly campanulate; Southern Ecuador and Peru: Cajamarca	**17**
17	Corolla scarlet red, tubular (beginning anthesis) to slightly campanulate (carpellate phase); sepals as long as petals; nectar scales with long dorsal filiform appendages to ca. 5 mm long; plants erect to subcandent up to 4 m long	***N.katjae* sp. nov.**
–	Corolla orange or bright red; sepals shorter than petals; nectar scales without dorsal filiform appendages, or these short, to ca. 1 mm long; plants less than 2 m tall	**18**
18	Corolla conical, petals tapering towards the apex, leaving only a small opening for the pollinator; sepals half as long as petals; flowers up to 4 cm long	***N.loxensis***
–	Corolla, cylindrical, petals straight, leaving an obvious opening for the pollinator; sepals ¾ as long as petals; flowers to ca. 2 cm long	***N.calycina* comb. nov.**

## Supplementary Material

XML Treatment for Nasa katjae

XML Treatment for Nasa calycina

XML Treatment for Nasa loxensis

## ﻿Additional information

### Author ORCIDs

Tilo Henning https://orcid.org/0000-0003-1929-3264

Joshua P. Allen https://orcid.org/0000-0001-6079-5261

Daniel Montesinos-Tubée https://orcid.org/0000-0002-4439-5089

Eric F. Rodríguez-Rodríguez https://orcid.org/0000-0003-0671-1535

José Luis Marcelo Peña https://orcid.org/0000-0002-0095-4643

Rafael Acuña-Castillo https://orcid.org/0000-0002-8568-8566

### Data availability

All of the data that support the findings of this study are available in the main text.
